# Nocturnal Leg Cramping Caused by Carnitine Deficiency Due to Long-Term Pivalate Antibiotics Administration in a Patient With Chronic Kidney Disease

**DOI:** 10.7759/cureus.48927

**Published:** 2023-11-16

**Authors:** Ryutaro Tanizaki, Yayoi Miyamatsu

**Affiliations:** 1 Internal Medicine and General Medicine, Ise Municipal General Hospital, Mie, JPN

**Keywords:** drug side effect, oral antibiotics, leg cramping, cefcapene-pivoxil, carnitine deficiency

## Abstract

Carnitine deficiency is a known cause of leg cramps and is sometimes observed in patients taking certain medications such as pivalate-containing antibiotics. A 69-year-old Japanese woman presented with a progression of painful involuntary nocturnal leg cramping. She had been taking cefcapene-pivoxil for six months. Serum-free carnitine (FC) and acylcarnitine levels were decreased. Then, carnitine deficiency due to long-term pivalate-containing antibiotics administration was diagnosed. After initiating oral L-carnitine treatment, her symptoms improved. It should be aware of carnitine deficiency if a patient taking pivalate-containing antibiotics presents with leg cramping.

## Introduction

Leg cramping is a common symptom reported by 50%-­60% of adults, and its prevalence increases with age [1]. While several causes of leg cramping, such as myopathic, neurologic, and metabolic factors, have been suggested, carnitine deficiency is also implicated in leg cramping.

Carnitine is an essential amino acid for fatty acid metabolism, as it is involved in transporting long-chain fatty acids into the mitochondrial matrix for beta-oxidation and removing excess fatty acids from the mitochondria as acylcarnitine (AC), resulting in ATP production [2]. In the normal course of muscle contraction, an increase in cytoplasmic calcium concentration is necessary. However, after contraction, ATP in the cytoplasm is expended, and the calcium ions are transported from the cytoplasm into the sarcoplasmic reticulum via a calcium pump. Consequently, the cytoplasmic calcium ion concentration decreases, leading to the cessation of muscle contraction. In the case of insufficient carnitine, the cytoplasmic calcium ion concentration remains elevated due to impaired ATP production, resulting in the persistence of muscle contraction and, subsequently, muscle cramps [3]. Leg cramping due to carnitine deficiency in adults is more common in patients undergoing peritoneal dialysis [4] and hemodialysis [5] and is mainly caused by insufficient dietary carnitine intake secondary to protein restriction and removal of carnitine from the blood as part of the dialysis process. In patients with non-dialysis chronic kidney disease (CKD), despite a restriction of protein intake and decreased biosynthesis of carnitine, free-carnitine (FC) levels do not drop with declining renal function, leading to reduced urinary excretion of carnitine relative to its intake [6].

Furthermore, carnitine deficiency can be caused by certain medications such as antiepileptic agents [7], particularly valproic acid [8], and pivalate-containing antibiotics [9]. Most reported cases of drug-induced carnitine deficiencies have involved children [9-11]. Herein, we describe a case of refractory nocturnal leg cramping caused by carnitine deficiency in a patient with long-term administration of pivalate-containing antibiotics.

## Case presentation

A 69-year-old Japanese woman with CKD and schizophrenia was referred to our hospital from a psychiatric clinic, with progression of anemia and intermittent, painful involuntary nocturnal leg cramping for several months. She denied exercise-associated muscle cramps. She had been administered aripiprazole, nitrazepam, and trazodone. Her symptoms of schizophrenia were well controlled, with no recent changes in antipsychotic medication. In addition, she had been taking cefcapene-pivoxil 100 mg three times daily for 6 months, prescribed by her psychiatrist to treat pyorrhea. Her leg cramping appeared 1 month after the administration of cefcapene-pivoxil.

On physical examination, she was afebrile, and her vital signs were unremarkable. Her leg skin findings, pulse palpation of the dorsalis pedis and popliteal arteries, and sensory nerve tests of the legs were normal. Neurological examination revealed no abnormalities including parkinsonism. She had no grasping pain in her legs.

Her initial laboratory test results showed no abnormalities, except normocytic anemia (hemoglobin, 6.8 g/dL; reference range: 9.0-14.0 g/dL) and decreased estimated glomerular filtration (eGFR) rate (28.1 mL/min/1.73 m^2^, normal range: 60 mL/min/1.73m^2^ or higher). Serum glucose was 101 mg/dL (normal range: 73-109 mg/dL). After admission, abdominal computed tomography, gastrointestinal endoscopy, and colonoscopy revealed no abnormalities. Additional blood tests revealed a Fe level of 15 mg/dL (reference range: 40-188 µg/dL), total iron binding capacity (TIBC) of 575 mg/dL (reference range: 250-450 µg/dL), and ferritin level of 4 ng/mL (reference range: 31-325 ng/mL). Subsequently, iron deficiency anemia and renal anemia were diagnosed, and intradermal erythropoietin and red blood concentrates were administered.

**Figure 1:**
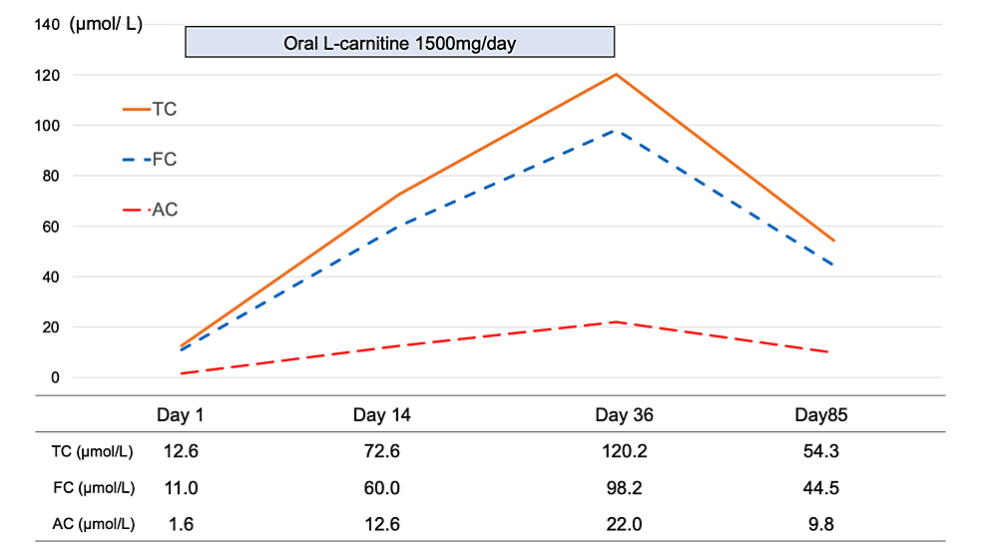
The transition of serum carnitine fraction The levels of TC, FC, and AC increased with the administration of L-carnitine. L-carnitine treatment was initiated on day 1. The AC/FC ratios on days 1, 14, 36, and 85 were 0.15, 0.21, 0.22, and 0.22, respectively. TC, total serum carnitine concentration; FC, serum free-carnitine concentration; AC, serum acylcarnitine concentration

Cefcapene-pivoxil was discontinued at admission; thereafter, serum total carnitine, FC, and AC levels were 12.6 µmol/L (reference range: 45-91 µmol/L), 11.0 µmol/L (reference range: 36-74 µmol/L), and 1.6 µmol/L (reference range: 6-23 µmol/L), respectively. The patient was diagnosed with carnitine deficiency, and oral L-carnitine (1,500 mg/day) treatment was initiated. As carnitine was administered, her symptoms gradually improved, and on day 7 after admission, she was discharged from the hospital. Fourteen days after starting L-carnitine treatment, her leg cramping frequency decreased to every alternate day, and the cramping had completely resolved by day 21. Oral L-carnitine treatment was discontinued on day 36 after confirming that the patient’s serum carnitine concentration had recovered to the normal range (Figure 1).

Since then, no recurrence of leg cramping has occurred in more than two years.

## Discussion

We described nocturnal leg cramping caused by carnitine deficiency which was induced by the administration of pivalate-containing antibiotics in a patient with CKD. In non-dialysis CKD patients, reduced selective urinary excretion of AC and impaired ß-oxidation lead to the accumulation of excess acyl groups [12]. To protect the mitochondria from toxic damage from excess acyl groups, FC is esterified to AC to buffer the excess acyl groups, resulting in increased AC levels and the resultant elevated AC/FC ratio, which indicates functional or relative carnitine deficiency [6]. In our patient, not only the serum FC level but also the AC concentration had decreased before L-carnitine treatment. The AC/FC ratio increased after the treatment and remained within the normal range (less than 0.4) [11] after discontinuing the treatment. This implies that the patient’s symptoms were caused by absolute carnitine deficiency related to the long-term administration of cefcapene-pivoxil. In addition, carnitine deficiency is associated with the progression of anemia due to the weakening of the erythrocyte membrane [13]. Thus, in our patient, in addition to iron deficiency anemia, the lack of erythropoietin, which is typical with renal anemia, along with carnitine deficiency, may have exacerbated the severity of anemia.

While iron deficiency anemia could cause leg cramping, it mainly occurs during exercise reflecting a lack of adequate oxygen supply to the muscles, and usually does not happen during nighttime sleep. In our patient, the leg cramping mainly occurred only at night, suggesting that the symptom was caused by carnitine deficiency rather than iron deficiency. Restless legs syndrome (RLS) is an important differential diagnosis in this case because it occurs mainly in the evening or at night when the patient is at rest and is sometimes accompanied by pain [14]. Additionally, iron deficiency is involved in exacerbating RLS symptoms [15]. However, RLS differs from leg cramps with patients experiencing more persistent discomfort and an urge to move their legs, with persistent muscle contractions not contributing to the symptoms. Furthermore, in this case, the leg cramp persisted even after correcting iron deficiency through blood transfusion, whereas the leg cramp did not occur after the blood carnitine concentration reached the normal range, supporting the diagnosis of leg cramping rather than RLS.

Neurological disorders play a crucial role in differentiating nocturnal leg cramping. Specifically, conditions such as motor neuron disease, neuropathy, radiculopathy, small-fiber sensory neuropathy, Parkinson's disease, and multiple sclerosis, can cause leg cramping [16]. However, in this case, there were no other clinical findings suggestive of these diseases, and the symptoms disappeared after carnitine supplementation, suggesting that carnitine deficiency alone can explain the cause of leg cramping. Other potential causes of nocturnal leg cramping, such as thyroid disease, tetany due to electrolyte abnormalities (e.g., hypocalcemia or hypomagnesemia), and drug-related factors [16], were not applicable in this patient.

Pivalate-containing antibiotics are converted to pivalic acid in the body, which binds to FC in the blood and is excreted as pivaloyl-carnitine in the urine, resulting in a decreased serum FC concentration [9]. Although the clinical symptoms of antibiotic-induced carnitine deficiency have been well documented in children [9-11], there have been fewer studies in adults. Our patient was administered the pivalate-containing antibiotic for the long term, which is a risk factor for the development of drug-induced carnitine deficiency. However, Kim et al. [17] described a 47-year-old woman with acquired encephalopathy associated with hypocarnitinemia a day after cefditoren-pivoxil administration. Furthermore, Hanai et al. [18] and Takahashi et al. [19] reported hypocarnitinemic hypoglycemia in an elderly man three and seven days after cefcapene-pivoxil administration, respectively. This suggests that even short-term administration of pivalate-containing antibiotics can result in carnitine deficiency. While hypocarnitinemic musculoskeletal symptoms such as muscle weakness and myalgia associated with pivalate-containing antibiotic administration have been reported [9], there are no reports of leg cramping involvement. To the best of our knowledge, this is the first adult case of leg cramping caused by carnitine deficiency associated with pivalate-containing antibiotics.

## Conclusions

Carnitine deficiency due to the administration of pivalate-containing antibiotics usually develops in children, although it can also be seen in adults. Leg cramping is a common symptom among the elderly, but it may be associated with carnitine deficiency. Drug-induced carnitine deficiency can be treated by discontinuing the respective drug and administering L-carnitine supplements. Physicians should be aware of carnitine deficiency in patients administered pivalate-containing antibiotics who present with leg cramping.
